# Economic evaluation: Impact on costs, time, and productivity of the incorporation of integrative digital pathology (IDP) in the anatomopathological analysis of breast cancer in a national reference public provider in Chile

**DOI:** 10.1016/j.jpi.2024.100417

**Published:** 2024-12-20

**Authors:** Rony Lenz-Alcayaga, Daniela Paredes-Fernández, Fancy Gaete Verdejo, Luciano Páez-Pizarro, Karla Hernández-Sánchez

**Affiliations:** a Universidad Andrés Bello – Public Health Institute, #700 Fernandez Concha Street, Las Condes, Santiago 7591538, Chile; b Santiago Oriente Dr. Luis Tisné Hospital - Pathology Department, #5150 Las Torres Avenue, Peñalolén, Santiago 7930124, Chile

**Keywords:** Digital pathology, Business process management, Breast cancer, Productivity, Costs

## Abstract

**Introduction:**

The incidence of breast cancer has risen in Chile, along with the complexity of diagnosis. For accurate diagnosis, it is necessary to complement the morphology assessed with hematoxylin and eosin with additional techniques to evaluate specific tumor markers. Evaluating the impact on costs, time, and productivity of automated techniques integrated with digital pathology solutions is crucial.

**Objectives:**

To estimate the impact on costs, time, and productivity of incorporating the automation of the HER2 in situ hybridization technique combined with integrative digital pathology (IDP) in breast cancer diagnosis in a Chilean public provider versus a manual technique.

**Methods:**

This economic evaluation adopted a health economics multi-method approach. A decision model was developed to represent the current manual fluorescence in situ hybridization (FISH) scenario versus an automated dual in situ hybridization (DISH) plus IDP in breast cancer diagnosis. Business process management (BPM) methodology was applied for capturing working time and latencies, in combination with a time-driven activity-based costing (TDABC) methodology for estimating direct, total, and average cost (2023 USD) for both scenarios for the following vectors: Human resources, supplies, and equipment, sorted by pre-analytical, analytical, and post-analytical phases. Indirect costs (2023 USD) were also retrieved. Both BPM and TDABC served to estimate labor productivity.

**Results:**

In the baseline scenario based on manual FISH, the turnaround time (TAT) was estimated at 1259 min, at an average total cost of $265.67, considering direct and indirect costs for all phases. An average of 20.5 FISH reports were submitted per pathologist monthly during the baseline. The automated DISH plus IDP scenario consumed 203 min per biopsy, at an average total cost of $231.08, considering direct and indirect costs for all phases; it also showed an average of 22.8 submitted reports per pathologist monthly. This represents a decrease of 13.02% in average total costs, an 83.86% decrease in TAT, and an average labor productivity increase of 11.29%.

**Conclusions:**

The incorporation of automated DISH plus IDP in the pathology department of this public provider has resulted in reductions in the time required to perform the in situ hybridization technique, a decrease in total costs, and increased productivity. Particular attention should be given to adopting new technologies to accelerate processing times and workflow.

## Highlights

• The incidence of breast cancer has risen in Chile, along with the complexity of its diagnosis.
• Automated HER2 DISH combined with IDP reduced time and costs, and improved productivity in a Chilean public provider.
• The baseline manual FISH turnaround time (TAT) was estimated at 1259 min, whereas the automated DISH+IDP TAT required 203 min (83.86% reduction).
• Total costs decreased by 13.02%, with an 11.29% increase in productivity.

## Introduction

The incidence of breast cancer has risen in Chile, along with the complexity of its diagnosis. In 2023, a total of 7503 women were diagnosed with breast cancer in the public sector.^1^ Nowadays, for an accurate cancer diagnosis, it is necessary to complement the morphology assessed with hematoxylin and eosin with additional techniques to evaluate specific tumor markers.

Charles Perou published the first molecular classification of breast cancer in 2000,^2^ leading to significant changes in oncology. Since then, molecular classification, molecular prognostic, and predictive factors have become essential in routine diagnosis to provide appropriate therapy, including molecular treatments. During the St. Gallen Consensus in 2013, it was agreed that the breast cancer molecular subtypes with therapeutic importance would be luminal A, luminal B, luminal HER2, pure HER2, and triple-negative (homologous to basal-like).^3^ These molecular subtypes can be approached by measuring four tumor markers: estrogen receptor (ER), progesterone receptor (PgR), HER2, and Ki67. HER2 is an oncogene on chromosome 17 encoding a transmembrane protein, a receptor belonging to the human epidermal growth factor receptor family, which is activated by gene amplification. Ki67 is a protein found only in dividing cells (proliferation marker). These four markers (ER, PgR, HER2, and Ki67) are diagnosed using immunohistochemical studies (IHC4) in most pathology departments nationwide. HER2 is confirmed with an additional molecular study using in situ hybridization (ISH). ISH techniques can rely on fluorescent in situ hybridization (FISH), dual in situ hybridization (DISH) (silver-based chromogenic technique), or chromogenic in situ hybridization (CISH) with a chromogen similar to that used in immunohistochemistry.^4^

In Chile, at the beginning of 2011, the Dr. Luis Tisné Brousse Santiago Oriente Hospital was defined as the national reference center for HER2 ISH in breast cancer, utilizing the manual FISH technique, which was the only method validated at that time as in vitro diagnostics by the U.S. Food and Drug Administration. This center continued using this technique until 2021 when a project was initiated to transition to an automated technique (DISH) integrated with digital pathology.

Several factors justify the implementation of technologies to improve pathology departments' productivity. Tumors account for 26.4% of deaths in Chile and are the leading cause of mortality nationwide.^5^ The National Health Authority reports age-adjusted cancer incidence rates that are projected to increase.^6^ Also, a growing demand for pathology services within the public healthcare provider network is predicted.^7^ Additionally, there is a shortage of physicians in Chile, coupled with their uneven geographical distribution.^8^

In Chile, no local cost studies have been conducted to estimate the average costs of breast cancer biopsies in the public sector. Additionally, there is a lack of research on how introducing novel technologies has impacted productivity in this field. Lack of costs and awareness of the benefits of Integrative Digital Pathology (IDP) have been acknowledged to lag its implementation in Latin America.^9^

In the context of increasing demand, it is urgent to elucidate the impact of this activity on public institutions, considering relevant cost drivers. The primary objective of this study was to estimate the impact on costs, time, and productivity of incorporating the automation of the HER2 ISH technique combined with IDP in breast cancer diagnosis, including automated equipment, slide scanners, pathology software, and artificial intelligence algorithms.

## Material and methods

This economic evaluation adopted a multi-method approach from the field of health economics. The study design involves measuring the baseline scenario and comparing it with a new scenario incorporating the new technology. Our estimations are based on a decision model and further costs, time, and productivity calculations. This economic evaluation is focused on the national reference public provider for the Oncology Network, Santiago Oriente Dr. Luis Tisné Brousse Hospital, which processes approximately 80% of the national public healthcare system demand for ISH HER2.

### Decision model

**Fig. 1:**
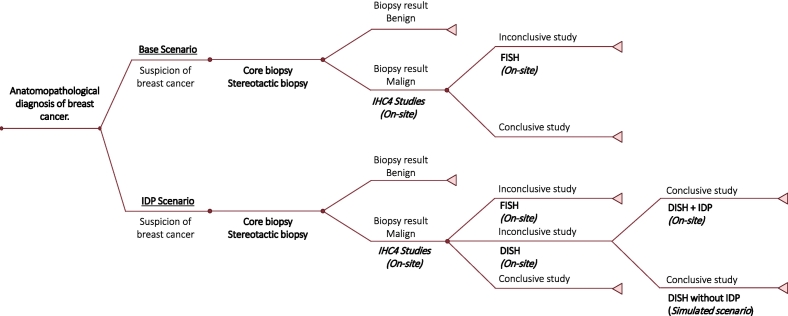
Decision model for estimating the impact on costs, time, and productivity of incorporating integrative digital pathology in the anatomopathological analysis of breast cancer. Source: From authors. The decision tree model displays the baseline scenario of manual FISH versus the analyzed intervention based on automated DISH in addition to integrative digital pathology (IDP).

A decision tree model was developed to systematically organize the pathology department's processes involved in biopsy analysis. The decision model for estimating the impact on costs, time, and productivity of incorporating IDP in the anatomopathological analysis of a breast cancer tumor marker (ISH HER2) is shown in Fig. 1.

The upper branch represents the baseline scenario of the ISH technique with fluorescence (FISH) using the manual method without incorporating IDP technology. In this branch, if breast cancer is detected, further analyses are needed to determine tumor markers. The routine tumor markers screened are ER and PgR, the HER2 oncogene, and Ki67. These markers are evaluated using IHC4 studies, and HER2 amplification is confirmed with a molecular ISH test. In Chile, 2+ and 3+ IHC4 cases require ISH confirmation.

The lower branch incorporates the effects of IDP to confirm the 2+ and 3+ immunohistochemical results using DISH with silver.

Based on data availability, the baseline was measured from July to November 2021 to avoid distortions due to COVID-19 peaks. During December 2021, the project included a validation phase to compare potential diagnostic variations between manual FISH (baseline scenario) and DISH plus IDP. Out of 50 analyses conducted, all reached a 100% diagnosis correlation. The IDP scenario was measured onsite from April to December 2022.

### Processes modeling

The processes were modeled using the value chain and process flowcharts methodology to analyze the stages needed for pathology diagnosis. This approach studies how work progresses within a healthcare organization.^10^ It sequentially describes the diagnostic process, identifies and visualizes task distribution, timing, critical nodes, and latencies, and analyzes stages for optimization. The business process management (BPM) methodology was employed for data gathering, utilizing descriptive techniques and vertical diagrams according to the American National Standards Institute. Flowcharts of the processes and subprocesses were utilized to detail working times, latencies, utilization rates, resources, inputs, and personnel responsible for each phase.

Fieldwork enabled us to identify and map these stages, which were subsequently organized into flowcharts and matrices. All of them are based on heterogeneous biochemical foundations (histochemistry, immunohistochemistry, ISH). The following set of activities have been described:

• Pre-analytical, analytical, and post-analytical phases for intraoperative biopsies.
• Analytical and post-analytical phases for deferred histopathological studies with hematoxylin-eosin staining.
• Pre-analytical, analytical, and post-analytical phases for deferred histopathological studies with immunohistochemistry techniques.
• Pre-analytical, analytical, and post-analytical phases for manual FISH and automated DISH with silver.

### Methodology for time-driven activity-based costing (TDABC)

One of the primary expected associated outcomes due to the intervention is the reduction of turnaround time (TAT), which refers to the elapsed time from receiving a sample in the pathology department until delivering the report with the diagnosis. Improvements in TAT can also increase productivity. We have adopted the time-driven activity-based costing (TDABC) methodology to measure time, costs, and resource utilization to solve cases during the entire cycle. TDABC also enables the identification and proposal of improvements to optimize complete cycles.^11^

#### Identification of average, total, direct, and indirect costs

We used a hybrid method combining statistical and expert-based analysis to estimate costs. Triangulation of different cost sources was considered, referencing processing manuals, expert experience, and hospital cost accounting systems. Productivity and cost drivers expressed in vertical formats were identified and validated.

The cost object corresponds to the anatomopathological diagnostic process of breast cancer samples (number of biopsies processed per unit of time). All subprocesses influenced by the evaluated technology have been costed.

Average costs correspond to total cost divided by the number of units produced. Therefore, at different production volumes, average costs will vary. This behavior is explained by the inverse relationship between costs and productivity and the decrease in fixed costs associated with increased production volume. Variable costs are those affected by the production level. On the other hand, fixed costs do not vary within a particular activity level or production volume. Total costs encompass the total resources incurred to generate different levels of production (variable costs + fixed costs), which can be broken down according to the productive resource (labor cost, input cost, capital costs, etc.). These costs can also be classified into direct and indirect costs as a second classification. Direct costs are directly related to the cost object, in this case, the anatomopathological diagnostic process of breast cancer samples. Indirect costs are those where a direct relationship between resource consumption and production cannot be established and are related to the support of the organization.^12^

We have combined bottom-up and top-down costing strategies in our analysis. The bottom-up strategy helped to identify all inputs required for the process, technical coefficients, and prices. This has been used to capture direct costs and itemize all components of the diagnostic process.^12^ The top-down strategy contributed to allocating expenditure to cost centers. It also allowed the estimation of average costs^12^ and indirect costs of this standardized process, as significant case-to-case variations were not expected.

Due to the differences in the observation periods for the scenarios compared in the decision model, all currencies have been adjusted to 2023 Chilean Pesos and converted to up-to-date U.S. dollars based on the Chilean Central Bank data.

### Sources of information for costing

#### Human resources

For this driver, we retrieved the following sources: Ordinary and extraordinary compensation of all employees in the pathology department (quarterly bonuses, year-end bonuses, overtime pay, vacation bonuses, customer service bonuses, and others) from payroll records (obtained from the Hospital's Human Resources Information System) for the last 12 months. We also estimated the contracted hours of each professional.

#### Indirect costs

Indirect costs were examined in WinSIG and PERC, implemented in Chile since 2000 as a requirement for self-managed hospitals. WinSIG and PERC are versions of a management information system developed by the Pan American Health Organization for decision-making and department-level costing.

#### Supplies and equipment

Prices of supplies from hospital tenders were examined. The purchasing prices of equipment (year of purchase and payment) for each piece are included. If the purchase price was not available, the replacement cost was explored. For rented or leased equipment, the monthly lease payment amount was required. We assumed the equipment cost is included in the corresponding service for equipment on loan. Also, the lifespan of each piece of equipment and the number of services to be executed per year were costed.

### Data validation strategy

Multiple instances of data validation were conducted, supported by comprehensive fieldwork documentation. This was crucial as our hybrid costing method combines statistical and expert elicitation. Exploratory visits were crucial to adapting the pathology department's procedural manual into detailed workflow flowcharts. The procedural manual focuses on technical details, whereas the flowcharts and costing matrices summarize the relationship between activities and their associated resource consumption. Time validations were conducted using manuals, expert technical assessments, and observations from researchers' visits.

## Results

Although our study encompassed the holistic estimations of costs and time for all techniques (including hematoxylin-eosin staining and IHC4), only the results of costs, time, and productivity of ISH HER2 are displayed in this publication for each scenario: Baseline manual FISH technique versus automated DISH technique in addition to IDP.

### Results of the manual FISH technique in the baseline scenario

#### Time associated with the manual FISH technique in the baseline scenario

**Fig. 2:**
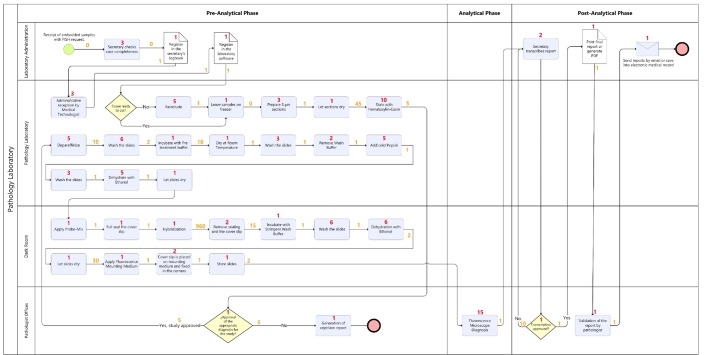
Process flow: Biopsy processing using manual fluorescence in situ hybridization (FISH) technique in the pathology department in baseline scenario. Source: From authors. Caption: Multi-channel process diagram showing the confluence of activities in the pathologist's office, dark room, pathology laboratory, and administration, according to pre-analytical, analytical, and post-analytical phases in manual FISH technique. The diagram represents activities, their respective latencies (numbers above connectors), and working time (inside boxes and rhombuses).

The following flowchart illustrates the activities needed for processing breast biopsies with the manual FISH technique (Fig. 2).

The pre-analytical phase required 105 min of working time. This increases when adding the incubation, storage, and hybridization time to 1222 min, or 20 hr (almost three working days). The analytical and post-analytical phases include the diagnosis using fluorescence microscopy up to the dispatch of signed reports. The total time for these phases was estimated at 37 min. The total time involved in developing the FISH technique was estimated at 127 min of working time. If latency and hybridization incubation times are included, the total time rises to 1259 min, or 21 hr, almost three working days (Table 1). Results for other techniques are also provided for reference.

**Table 1:**
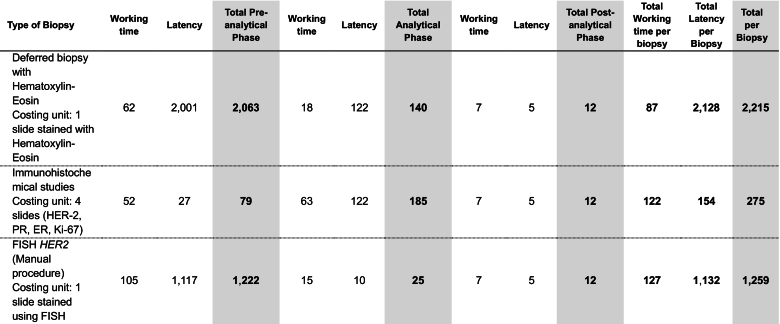
Total turnaround time (TAT in minutes) per biopsy type, process stage, and type of activity in the baseline scenario.

Bold text signifies sub-totals and totals. For instance, in the "Total Pre-Analytical Phase," the sub-total is calculated by summing its components: working time and latency. Similarly, the "Total Analytical Phase" is derived by adding the working time and latency specific to that phase. This structure is consistent across all phases (pre-analytical, analytical, and post-analytical), where working time and latency collectively contribute to the total time for each phase. In the case of the columns labeled "Total Working Time per Biopsy," "Total Latency per Biopsy," and "Total per Biopsy," these represent final aggregated estimates, consolidating the respective components into a comprehensive measure for each biopsy.

#### Direct unit costs of manual FISH technique in baseline scenario

The direct unit costs correspond to human resources, supplies, and equipment. The FISH technique has been presented continuously from the perspective of resource consumption, from reception to report dispatch. For the human resources driver, the cost with the highest share corresponds to the microscopic diagnosis milestone, comprising one-third of the total unit cost of this category (Table 2).

**Table 2:** Unit cost of human resources (H.R.) for FISH technique in the baseline scenario.

Milestone	Professional	Utilization rate	Hourly wage	Time (minutes)	Wage per minute	Total H.R. vector
Checking case completeness	Secretary	1	$6.04	3	$0.10	$0.30
Register in the secretary's logbook	Secretary	1	$6.04	1	$0.10	$0.10
Reinclude (if necessary)	Lab technician	0.2	$15.13	5	$0.25	$0.25
Storage of samples in the freezer	Lab technician	1	$15.13	1	$0.25	$0.25
Prepare 3 μm sections	Lab technician	1	$15.13	3	$0.25	$0.76
Let sections dry	Lab technician	1	$15.13	1	$0.25	$0.25
Stain 1 slide with hematoxylin-eosin	Lab technician	1	$15.13	10	$0.25	$2.52
Approve diagnosis to proceed with ISH technique	Pathologist	1	$45.45	1	$0.76	$0.76
Deparaffinize	Lab technician	1	$15.13	5	$0.25	$1.26
Wash slides	Lab technician	1	$15.13	6	$0.25	$1.51
Incubate with pre-treatment buffer	Lab technician	1	$15.13	1	$0.25	$0.25
Dry at room temperature	Lab technician	1	$15.13	1	$0.25	$0.25
Wash slides	Lab technician	1	$15.13	3	$0.25	$0.76
Remove wash buffer	Lab technician	1	$15.13	2	$0.25	$0.50
Add cold pepsin	Lab technician	1	$15.13	5	$0.25	$1.26
Wash slides	Lab technician	1	$15.13	3	$0.25	$0.76
Dehydration with ethanol	Lab technician	1	$15.13	5	$0.25	$1.26
Dry the slides	Lab technician	1	$15.13	1	$0.25	$0.25
Apply probe-mix	Lab technician	1	$15.13	1	$0.25	$0.25
Seal the slides	Lab technician	1	$15.13	1	$0.25	$0.25
Place the slides in the hybridizer	Lab technician	0.05	$15.13	1	$0.25	$0.01
Remove sealing	Lab technician	1	$15.13	2	$0.25	$0.50
Incubate with stringent wash buffer	Lab technician	1	$15.13	1	$0.25	$0.25
Wash slides	Lab technician	1	$15.13	6	$0.25	$1.51
Dehydration with ethanol	Lab technician	1	$15.13	6	$0.25	$1.51
Dry the slides	Lab technician	1	$15.13	1	$0.25	$0.25
Apply fluorescence mounting medium	Lab technician	1	$15.13	1	$0.25	$0.25
Seal and label the slides	Lab technician	1	$15.13	2	$0.25	$0.50
Store the slides	Lab technician	1	$15.13	1	$0.25	$0.25
Microscopic diagnosis	Pathologist	1	$45.45	15	$0.76	$11.36
Register on laboratory information system	Secretary	1	$6.04	2	$0.10	$0.20
Transcription approval	Pathologist	1	$45.45	1	$0.76	$0.76
Print the report	Secretary	1	$6.04	1	$0.10	$0.10
Report validation	Pathologist	1	$45.45	1	$0.76	$0.76
Send report	Secretary	1	$6.04	1	$0.10	$0.10
Total H.R. cost						**$32.11**

Note: Please consider variations in the estimates due to the effects of currency conversion and the precision of decimal values.

Due to a loan-for-use commercial agreement, the FISH HER2 bundle represents 82.89% of the direct cost of this technique. The bundle includes equipment, supplies, and reagents for performing the technique. Hospitals using other purchasing forms should be cautious when extrapolating the herby analysis to their setting. Details are provided in Table 3 and Table 4.

**Table 3:** Unit cost of supplies for manual FISH technique in the baseline scenario.

Supplies	Utilization rate	Coverage	Unit of measure	Unit cost	Purchase quantity	Purchase price	Conversion unit	Total supplies vector
Supplies per FISH								
FISH HER2 bundle	1	100%	1 assay	$161.56	1700 assays/year	$274,652.25	1	$161.56
Batch supplies								
Procedure gloves	1	100%	1 pair	$0.21	100 boxes of 100 units	$1044.03	2	$0.21
Xylol	6	100%	1 biopsy	$0.04	1560 l	$11,322.47	200	$0.22
Ethanol absolute	6	100%	1 biopsy	$0.04	2250 l	$18,214.77	200	$0.24
Total supplies vector								**$162.23**

Note: Please consider variations in the estimates due to the effects of currency conversion and the precision of decimal values.

**Table 4:** Unit cost of equipment for manual FISH technique in the baseline scenario.

Equipment^a^	Utilization rate	Acquisition cost	Lifetime	Annual cost	Tests per year	Operating hours per day	Total equipment vector
Freezer (−20 °C)	1	$7941.92	9 years	$882.44	1831	8	$0.48
Refrigerator (2–8 °C)	1	$1605.13	9 years	$178.35	1831	8	$0.10

a The herby-reported equipment is additional to the equipment considered in the FISH HER2 bundle due to the loan-for-use commercial agreement. Please consider variations in the estimates due to the effects of currency conversion and the precision of decimal values.

#### Total unit cost of the manual FISH technique in the baseline scenario

An average total cost of $265.67 was estimated, considering direct and indirect costs (Table 5). The major cost component can be attributed to the pre-analytical phase (93.19%) and supplies (61.06%).

**Table 5:** Summary table of total unit costs for the manual FISH technique in the baseline scenario.

Resources	Pre-analytical phase		Analytical phase		Post-analytical phase		Total	%
	Costs	%	Costs	%	Costs	%		
Human resources	$18.83	58.64%	$11.36	35.39%	$1.92	5.97%	$32.11	**12.09%**
Supplies	$162.23	100%	$0.00	0.00%	$0.00	0.00%	$162.23	**61.06%**
Equipment	$0.58	100%	$0.00	0.00%	$0.00	0.00%	$0.58	**0.22%**
Average direct unit cost	**$181.64**	**93.19%**	**$11.36**	**5.83%**	**$1.92**	**0.98%**	**$194.92**	**73.37%**
Average indirect unit cost^a^							**$70.76**	**23.63%**
Average total unit cost							**$265.67**	**100%**

a Allocating a portion of the cost center's indirect costs to each specific biopsy type was necessary to estimate indirect costs related to breast cancer biopsies, resulting in a 26.63% indirect cost out of total costs for these biopsies. Please consider variations in the estimates due to the effects of currency conversion and the precision of decimal values.

### Productivity in the baseline scenario

The average labor productivity in the baseline scenario consists of the ratio between production (output) and the human resources to produce it. The average productivity of medical work is presented. Production was assessed over 5 months. The labor of the physicians was analyzed for the same period and adjusted to a standard 44-hr workweek for subsequent comparison.

The staff consisted of eight pathologists, with their respective weekly hours detailed in Table 6. The equivalent number of full-time contracts, based on a 44-hr workweek, amounts to 6.5. During the study period (baseline), an average of 20.5 FISH reports were submitted monthly per pathologist (adjusted by equivalent full-time contracts).

**Table 6:** Average labor productivity associated with the manual FISH technique in the baseline scenario.

Physician	Weekly hours	Monthly hours	FISH performed
Pathologist n° 1	44	176	**799**
Pathologist n° 2	44	176	
Pathologist n° 3	44	176	
Pathologist n° 4	44	176	
Pathologist n° 5	33	132	
Pathologist n° 6	22	88	
Pathologist n° 7	22	88	
Pathologist n° 8	33	132	
Total weekly hours			**1144**
Equivalent adjusted full-time contracts			**6.5**
Adjusted average labor productivity (FISH reports per equivalent full-time contracts per month per pathologist)			**20.5**

### Results of the automated DISH technique in addition to integrative digital pathology scenario

In this project, two techniques were automated: DISH and the diagnosis based on previous DISH using IDP. The following results are focused on IDP.

IDP has the potential to improve the pathology department's productivity. Since 2022, the pathology department of the Santiago Oriente Dr. Luis Tisné Brousse Hospital has implemented a set of technological solutions known as IDP by Roche. IDP comprises a slide scanner VENTANA DP-200, the *uPath* management software, and the integration of artificial intelligence-assisted analysis algorithms applied to the previous image obtained.

#### Times associated with the automated DISH technique in addition to integrative digital pathology (IDP) scenario

**Fig. 3:**
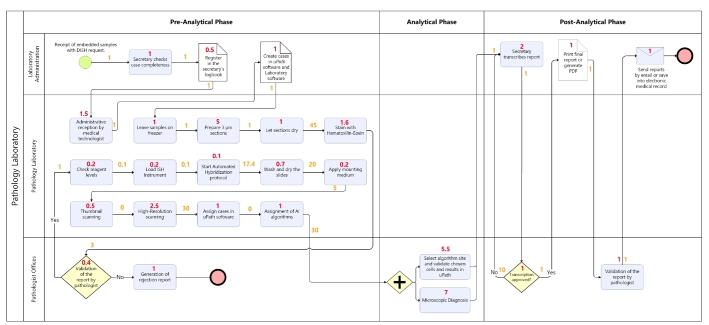
Process flow: Biopsy processing via automated DISH technique in addition to integrative digital pathology (IDP) in the pathology department. Source: From authors. Caption: Multi-channel process diagram showing the confluence of activities in the pathologist office, pathology laboratory, and administration according to pre-analytical, analytical, and post-analytical phases in an automated DISH + IDP scenario. The diagram represents activities, their respective latencies (numbers above connectors), and working time (inside boxes and rhombuses).

The IDP process begins once the Benchmark Ultra protocol for DISH HER2 has been completed and the coverslips have been mounted. In the secretary's office, cases (patient's name, identifier number, and test number) are created using the uPath software. Once the cases are created, the images are digitized from the slides on the VENTANA DP-200 scanner, which has a capacity for six slides and is connected to a server that allows remote storage and analysis. Subsequently, the digitized images are assigned to the corresponding case and made available for pathologists (Fig. 3).

This scenario leads to reductions in the needed time to issue reports. Automated DISH combined with IDP consumes 203 min per biopsy (reduction of −83.86%), distributed as 33 min of working time (reduction of −74.18%) and 170 min of latency (reduction of −84.94%). This reduction in total time is mainly achieved by reducing latencies (−84.94%) and reducing the time involved in the pre-analytical phase of the process (−84.32%). These results are presented in Table 7.

**Table 7:** Total turnaround time (TAT in minutes) per biopsy type, process stage, and type of activity comparing the baseline scenario versus automated DISH technique in addition to integrative digital pathology (IDP).

Type of biopsy	Working time	Latency	Total pre-analytical phase	Working time	Latency	Total analytical phase	Working time	Latency	Total post-analytical phase	Total working time	Total latency	Total
FISH *HER2*	105	1117	**1222**	15	10	**25**	7	5	**12**	**127**	**1132**	**1259**
DISH *HER2*/Cen17 + IDP	21	170	**192**	7	0	**7**	5	0	**5**	**33**	**170**	**203**
Difference (minutes)	−84	−947	**−1030**	−9	−10	**−19**	−2	−5	**−7**	**−94**	**−962**	**−1056**
Difference (%)	−79.84%	−84.74%	**−84.32%**	−56.67%	−100%	**−74.00%**	−23.08%	−100%	**−56.52%**	**−74.18%**	**−84.94%**	**−83.86%**

Note: Please consider variations in the estimates due to the effects of decimal values.

Readers should consider that DISH could be used without IDP as well. We conducted a sensitivity analysis to simulate the effects of the automation in the absence of IDP (Table 8). The data show an 18.56% increase in total processing time when IDP is used. We observe a 47.03% increase in the pre-analytical phase, mainly for lab technicians (due to incorporating new steps such as slide scanning). However, time decreases by 63.89% and 78.26% in the analytical and post-analytical phases because of automation and reduced times for the pathologist.

**Table 8:** Sensitivity analysis for estimating total turnaround time (TAT in minutes) in automated DISH technique with and without the addition to integrative digital pathology (IDP).

Type of biopsy	Working time	Latency	Total pre-analytical phase	Working time	Latency	Total analytical phase	Working time	Latency	Total post-analytical phase	Total working time	Total latency	Total
DISH *HER2*/Cen17 + IDP	21	170	**192**	7	0	**7**	5	0	**5**	**33**	**170**	**203**
Sensitivity analysis: DISH *HER2*/Cen17 without IDP	19	111	**130**	8	10	**18**	11	12	**23**	**38**	**133**	**171**
Difference (minutes)	**2**	**59**	**61**	**−2**	**−10**	**−12**	**−6**	**−12**	**−18**	**−5**	**37**	**32**
Difference (%)	**12.18%**	**52.93%**	**47.03%**	**−18.75%**	**−100%**	**−63.89%**	**−54.55%**	**−100%**	**−78.26%**	**−13.74%**	**27.72%**	**18.56%**

Note: Please consider variations in the estimates due to the effects of decimal values.

#### Direct unit costs of the automated DISH technique in addition to integrative digital pathology (IDP) scenario

In the analytical phase, pathologists diagnose using the *uPath* software, which allows remote access to the case study, access to the scanned tissue, and determination of the ratio between different signals generated by the division of the HER2 gene/Chromosome 17 Centromere. This process takes an average of 5.5 min and has an estimated cost of $4.17, depending entirely on the sample's complexity, the patient's clinical characteristics, and the product quality prepared in the pre-analytical phase. This justifies performing the diagnosis without artificial intelligence assistance in approximately one out of every seven cases, which takes an average of 7 min, as some cases require a more detailed review. Considering these proportions, the analytical phase has an estimated cost of $4.92 and a duration of 7 min. Finally, in the post-analytical phase, which begins when the pathologist completes the diagnosis, the report is generated and validated and later distributed in PDF format to the requestors. This stage has a direct cost attributable to human resources amounting $3.13 (Table 9, Table 10).

**Table 9:** Unit cost of human resources (H.R.) for the pre-analytical, analytical, and post-analytical phase of breast cancer biopsy processing via automated DISH technique in addition to integrative digital pathology (IDP).

	Professional	Utilization rate	Hourly wage	Time (minutes)	Wage per minute	Total H.R. vector

*Pre-analytical phase milestone*						

ISH request reception	Secretary	1	$6.04	1	$0.10	$0.10
Receipt of samples	Lab technician	1	$15.13	1.5	$0.25	$0.38
Create the case in *uPath* software	Secretary	1	$6.04	1	$0.10	$0.10
Leave samples in the freezer	Lab technician	1	$15.13	1	$0.25	$0.25
Prepare 3 μm sections	Lab technician	1	$15.13	5	$0.25	$1.26
Let sections dry	Lab technician	1	$15.13	1	$0.25	$0.25
Stain with hematoxylin-eosin	Lab technician	0.75	$15.13	2.14	$0.25	$0.41
Pathologist approves diagnosis	Pathologist	0.75	$45.45	0.5	$0.76	$0.28
Check reagent levels	Lab technician	0.03	$15.13	5	$0.25	$0.04
Load ISH instrument	Lab technician	0.03	$15.13	5	$0.25	$0.04
Start hybridization protocol	Lab technician	0.03	$15.13	0.5	$0.25	$0.00
Wash and dry the slides	Lab technician	0.03	$15.13	20	$0.25	$0.17
Apply mounting medium	Lab technician	0.03	$15.13	5	$0.25	$0.04
Thumbnail scanning	Lab technician	1	$15.13	0.5	$0.25	$0.13
High-resolution scanning	Lab technician	1	$15.13	2.5	$0.25	$0.63
Assign cases	Lab technician	1	$15.13	1.0	$0.25	$0.25
Operation of A.I. algorithms	Lab technician	1	$15.13	1	$0.25	$0.25
Check the cases	Pathologist	1	$45.45	2.5	$0.76	$1.89
Total H.R. vector pre-analytical phase						**$6.49**


*Analytical phase milestone*						

Diagnosis using *uPath*	Pathologist	1	$45.45	5.5	$0.76	$4.16
Microscopic diagnosis	Pathologist	0.14	$45.45	7.0	$0.76	$0.76
Total H.R. vector analytical phase						**$4.92**


*Post-analytical phase milestone*						

Generation and validation of final report	Pathologist	1	$45.45	4	$0.76	$3.03
Send report	Secretary	1	$6.04	1	$0.10	$0.10
Total H.R. vector post-analytical phase						**$3.13**

Note: Please consider variations in the estimates due to the effects of currency conversion and the precision of decimal values.

**Table 10:** Unit cost of supplies for the automated DISH technique in addition to integrative digital pathology (IDP).

Supplies	Utilization rate	Coverage	Unit of measure	Unit cost	Purchase quantity	Purchase price	Conversion unit	Total supplies vector
DISH bundle	1	100%	1 assay	$161.70	1500 assays/year	$232,065.64^a^	1	$154.71
Procedure gloves	1	100%	1 pair	$0.04	100 boxes of 100 units	$925.79	2	$0.19
Total supplies vector								**$154.90**

a Price based on specifications in tender 1,057,492-118-LQ21 from 2022 refers to equipment on loan and reagents; therefore, the cost will be allocated to the supplies item. This bundle includes the equipment for performing DISH (Benchmark Ultra), the automated image digitization system (Ventana DP-200), the *uPath* computer storage/query system, and the diagnostic algorithm system for DISH *HER2*. Hospitals using other purchasing forms should be cautious when extrapolating the herby analysis to their setting. Please consider variations in the estimates due to the effects of currency conversion and the precision of decimal values.

#### Total unit costs of automated DISH technique in addition to integrative digital pathology (IDP) scenario

Average total unit cost of automated DISH combined with IDP was $231.04.Total direct costs account for 73.37% ($169.54). The unit cost of human resources is 44.61% ($6.49) in the pre-analytical phase, 33.86% ($4.92) in the analytical phase, and 21.53% ($3.13) in the post-analytical phase. The pre-analytical phase accounts for 95.2% ($161.48) of direct costs (Table 11).

**Table 11:** Summary table of total unit costs for the automated DISH technique in addition to integrative digital pathology (IDP) scenario.

Cost driver	Pre-analytical phase		Analytical phase		Post-analytical phase		Total	%
	Cost	%	Cost	%	Cost	%		
Human resources	$6.49	44.61%	$4.92	33.86%	$3.13	21.53%	**$14.54**	**6.29%**
Supplies	$154.90	100%	$0.00	0.00%	$0.00	0.00%	**$154.90**	**67.03%**
Equipment	$0.10	100%	$0.00	0.00%	$0.00	0.00%	**$0.10**	**0.04%**
Average unit direct cost	**$161.48**	**95.2%**	**$4.92**	**2.9%**	**$3.13**	**1.8%**	**$169.54**	**73.37%**
Average unit indirect cost							**$61.50**	**26.61%**
Average total unit cost							**$231.04**	**100%**

Note: Please consider variations in the estimates due to the effects of currency conversion and the precision of decimal values.

### Summary comparisons between manual FISH baseline scenario and automated DISH technique in addition to integrative digital pathology (IDP) scenario

The average difference in the total unit cost between the baseline scenario ($265.67) and the scenario with automated DISH in addition to IDP ($231.08) is −13.02%.

The TAT in the manual FISH baseline scenario was estimated at 1259 min, compared to 203 min in the implemented scenario with automated DISH plus IDP, representing a total time saving of 83.86% (74.18% decrease in working time and 84.94% decrease in latency). The most significant change is observed in the pre-analytical phase, with an 84.32% reduction in time. Analytical and post-analytical phases also exhibit changes (−74.00% and −56.52%, respectively).

Production (output) in the baseline scenario was 799 reports. To accurately interpret changes in production—the number of samples processed between the two scenarios—note that the output of the pathology department operates on a pro re nata basis, meaning it is driven by demand from the hospital and other centers submitting samples. Therefore, it depends not only on increases in staffing or new capital investments but also on the quantity of samples received. After incorporating the automated DISH + IDP technique, the output increased to 855 reports (7.01% increase).

Implementing automated DISH combined with IDP, has increased the adjusted average medical labor productivity by 11.29% to date, at an early learning curve (Table 12)

**Table 12:** Summary comparison between manual FISH baseline scenario and automated DISH technique in addition to integrative digital pathology (IDP) scenario for costs, time, and labor productivity.

Parameter	Manual FISH scenario	Automated DISH + IDP scenario	Variation (%)
Average unit direct cost per biopsy in 2023 USD	$194.92	$169.54	**−13.02%**
Average direct + Indirect costs (total unit cost) per output in 2023 USD	$265.67	$231.08	**−13.02%**
Production in the studied period (output)	799	855	**+7.01%**
Turnaround time (TAT) measured in minutes	1259	203	**−83.86%**
Pre-analytical TAT (min)	1222	192	**−84.32%**
Analytical TAT (min)	25	7	**−74.00%**
Post-analytical TAT (min)	12	5	**−56.52%**
Adjusted average labor productivity (FISH reports per equivalent full-time contracts per month per pathologist)	20.5	22.8	**+11.29%**

Note: Please consider variations in the estimates due to the effects of currency conversion and the precision of decimal values.

## Discussion

Using BPM in healthcare is crucial to establishing an interface between clinical and management teams. However, its adoption in healthcare has progressed slowly. Pufahl *et al*. (2022) explored patient-related, medical practice-specific, and medical resource-related challenges.^10^ Evidence supports the positive impact of BPM on the management and optimization of clinical processes. However, in function-oriented providers, BPM lacks value as an informative instrument. Also, in systems without abundant digitalized information, using BPM may require simulation and qualitative data, adding potential bias to the estimations.^13^ Nonetheless, that approach fails to acknowledge the role of experts as sources of valid information. Evidence acknowledges that BPM alone does not structure process-based management. The use of BPM must be accompanied by in-depth processes that integrate technology, organization, and people^14^.

The methodology used in our study combines process mapping, activity-based costing, and time-driven activity-based costing as innovations in costing methods applied in this field. We could elaborate flowcharts and cost matrixes based on local process workflows and national guidelines for sample processing.

Based on these results, the financial impact of transitioning from manual FISH to automated DISH combined with the IDP, is practically neutral. This is positive from the perspective of average labor productivity because these improvements expand production, significantly reduce TAT, and liberate resources to increase the pathology department's production frontier.

Incorporating automated DISH combined with IDP resulted in changes in the workflow, as predicted by Bruce *et al*. (2024).^15^ Some studies have measured differences in TAT due to the implementation of digitalization innovations in pathology departments. In the Canadian experience, the times associated with robotic microscopy and virtual slide telepathology systems are 19.98 min and 15.68 min, respectively (*p* < 0.0001). With the implementation of virtual slide telepathology technology, these times are explained by a 4-fold decrease in slide interpretation time.^16^ According to Vergani *et al*. (2018), the TAT between the conventional analysis method and those associated with digital pathology vary from a median of 12 days (range 3–45 days) to a median of 1.4 days (range 1–2 days).^17^ Retamero *et al*. (2020) found a similar trend during the transition to complete digital pathology for routine histopathology diagnosis in four centers in Spain. The authors estimated that the benefits of these technologies are based on gains in efficiencies in the pre-analytical and analytical stages, as well as the support these technologies provide for assisted diagnosis.^18^ In the Netherlands, digitizing the pathology department demonstrated a general decrease in TAT, from 6.16 days to 5.73 days (6.94%). The most significant effect was observed in the categories of cases considered complex. In the most complex subgroup, the TAT decreased from 9.66 days to 7.72 days (20.16%).^19^ According to Baidoshvili *et al*. (2018), digital pathology saves time during case preparation. These savings in total are equivalent to 1147 min per day.^20^

The experiences consistently show efficiency gains associated with digital innovations. Authors in studies involving deep learning assistance algorithms in breast cancer emphasize that the average review time per image is significantly shorter with A.I. This is observed for both micrometastases (average review time per image with assistance was 61 sec versus 117 sec without assistance, *p* = 0.002) and negative images (111 sec versus 136 sec, *p* = 0.018).^21^ A comparative study of 400 cases showed shorter times with digital pathology (1841 min) compared to optical microscopy (1956 min).^22^

The reduction in TAT allows for an increase in output capacity. One of the elements contributing to cost stability and reduced TAT is the increase in medical labor productivity (11.29%), as the number of examinations per physician per month rises from 20.5 to 22.8. This increase is attributed to a 7.01% increase in output. Also, it is attributed to a decrease (−4%) in the number of equivalent full-time physicians (more efficient staff). Although implementing IDP improved the productivity of pathologists, it did not affect lab technicians' productivity. On the other hand, the automation of the ISH technique improved the productivity of lab technicians. This automation did not impact the pathologists. Our experience is consistent with the findings of improved productivity in other publications. Griffin and Treanor (2017), based on the model from Ho *et al*. (2014), estimated that 10% or 15% productivity improvements would reach a breakeven point in the first and second year, respectively, resulting in a net benefit due to the introduction of digital pathology. Griffin and Treanor (2017) stated that a department half the size described by Ho *et al*. (2014) would achieve gains after 4 years with a 10% productivity improvement.^23^ Retamero *et al*. indicate that the number of histology cases per pathologist increased by 17% in 2016, 20% in 2017, and 26% in 2018 compared with the year before going fully digital. Additionally, on average, the pathologists have signed out 21% more reports each year since implementing full digital pathology for primary diagnosis.^18^

The change in unit costs indicates that the new technology can be incorporated without cost pressures. Our data suggest that the average cost of automated DISH in addition to IDP, decreases by 13.02% compared to the manual FISH technique. Ho *et al*. (2014), based on an expected 13% productivity increase associated with digital pathology, estimated that savings could exceed $17.7 million in breast cancer and melanoma over a 5-year horizon. The most significant savings come from productivity benefits ($12.4 million), with an additional $5.4 million deriving from quality improvements. These improvements are also supported by the technology's ability to increase medical productivity and facilitate workload redistribution. As a result, pathologists would increase their output by 1.5 cases per workday.^24^ Similarly, Hanna *et al*. (2020) projected annual savings exceeding $267,000. These savings are attributed to personnel restructuring, reduced vendor services, and savings on physical storage, among others.^25^ Additional studies extrapolate professional time savings, estimating that saving approximately 19 hr daily is equivalent to €120,000 per year.^20^

Similarly, from a qualitative perspective, the introduction of digital pathology is well perceived by professionals, who point out benefits in training, including easier access to cases to enhance individual learning and teaching.^26^

As part of the limitations of this study, the post-implementation measurement was conducted early in the learning curve and during the initial stages of clinical team adherence. This was due to constraints imposed by the project timeline. This may have led to underestimating the effects on costs, TATs, and productivity. Additionally, scenarios involved different combinations of technologies, generating a combined effect leading to joint efficiency gains in the second scenario. The last explains the need for the sensitivity analysis presented above. These factors are related to the introduction format of the technology agreed between the hospital and the technology producer rather than a design issue of our economic evaluation.

Additional limitations are grounded on the lack of well-defined methodologies for differentiated economic evaluations of medical devices incorporating digital technologies in Chile. In general, health technology assessments of digital technologies follow the recommendations used to evaluate molecules. However, digital technologies entail different metrics, benefits, outcomes, and costs from those needed for pharmaceuticals. The National Institute for Health and Care Excellence acknowledges some of these particularities. In this study, we have applied the standards for digital health technologies assessment by describing the current and proposed pathways, outlining the expected impacts on healthcare, cost, and resources, providing evidence of the effectiveness of digital technology (TAT as a surrogate), and demonstrating real-world evidence that the claimed benefits can be achieved in practice.^27^

## Conclusion

Incorporating automated DISH combined with IDP in the pathology department of Santiago Oriente Dr. Luis Tisné Brousse Hospital has reduced time and total costs, and increased productivity. Automated DISH in addition to IDP has demonstrated to be a financially efficient technique, with the potential to liberate capacities for staff to engage in additional activities. Adopting this new technique under these parameters is an efficiency driver for the pathology department.

This has become the first TDABC study on breast cancer biopsies in the public sector that incorporates integrative digital technologies in Chile. Due to its impact, special attention should be given to innovation in adopting new technologies to accelerate processing times and workflow.

As part of the socialization process of these results, a stakeholders meeting was held in October 2023 with Ministry of Health representatives, public and private providers, and national health services leaders. In 2024, the National Cancer Plan for Adults has considered strengthening the integration of the oncology provider network. A desirable action is implementing a digital pathology network that enables expert consultations at the national level.^28^

## Financing source

This work was supported by 10.13039/100004337Roche Chile. Roche had no participation in the formulation of methods, interpretation of data, nor publication.

## Declaration of competing interest

Professor Rony Lenz reports financial support, article publishing charges, and travel for congress presentation provided by Roche. Dr. Fancy Gaete reports that Roche provided financial support for travel for congress presentation purposes only. Professor Daniela Paredes, Luciano Páez, and Karla Hernández declare that they have no known competing financial interests or personal relationships that could have appeared to influence the work reported in this article.
